# Evidence of Recombination in Intrapatient Populations of Hepatitis C Virus

**DOI:** 10.1371/journal.pone.0003239

**Published:** 2008-09-18

**Authors:** Vicente Sentandreu, Nuria Jiménez-Hernández, Manuela Torres-Puente, María Alma Bracho, Ana Valero, María José Gosalbes, Enrique Ortega, Andrés Moya, Fernando González-Candelas

**Affiliations:** 1 Institut Cavanilles de Biodiversitat i Biologia Evolutiva, Universitat de València Valencia, Spain; 2 CIBER Epidemiología y Salud Pública (CIBERESP), Barcelona, Spain; 3 Unidad de Enfermedades Infecciosas, Hospital General Universitario, Valencia, Spain; The Rockefeller University, United States of America

## Abstract

Hepatitis C virus (HCV) is a major cause of liver disease worldwide and a potential cause of substantial morbidity and mortality in the future. HCV is characterized by a high level of genetic heterogeneity. Although homologous recombination has been demonstrated in many members of the family Flaviviridae, to which HCV belongs, there are only a few studies reporting recombination on natural populations of HCV, suggesting that these events are rare *in vivo*. Furthermore, these few studies have focused on recombination between different HCV genotypes/subtypes but there are no reports on the extent of intra-genotype or intra-subtype recombination between viral strains infecting the same patient. Given the important implications of recombination for RNA virus evolution, our aim in this study has been to assess the existence and eventually the frequency of intragenic recombination on HCV. For this, we retrospectively have analyzed two regions of the HCV genome (NS5A and E1-E2) in samples from two different groups: (i) patients infected only with HCV (either treated with interferon plus ribavirin or treatment naïve), and (ii) HCV-HIV co-infected patients (with and without treatment against HIV). The complete data set comprised 17712 sequences from 136 serum samples derived from 111 patients. Recombination analyses were performed using 6 different methods implemented in the program RDP3. Recombination events were considered when detected by at least 3 of the 6 methods used and were identified in 10.7% of the amplified samples, distributed throughout all the groups described and the two genomic regions studied. The resulting recombination events were further verified by detailed phylogenetic analyses. The complete experimental procedure was applied to an artificial mixture of relatively closely viral populations and the ensuing analyses failed to reveal artifactual recombination. From these results we conclude that recombination should be considered as a potentially relevant mechanism generating genetic variation in HCV and with important implications for the treatment of this infection.

## Introduction

Hepatitis C virus (HCV) infection affects about 170 million people worldwide, about 3% of the world's population [1], and is the major cause of liver disease and a potential cause of substantial morbidity and mortality in the future [2]. Hepatitis C virus is the only species of the genus *Hepacivirus* within the family *Flaviviridae*. It has a single stranded, positive-sense, nonsegmented RNA genome of about 9600 nucleotides (nt) with a single, long open reading frame encoding a polyprotein of about 3000 amino acids with the gene order C-E1-E2-p7-NS2-NS3-NS4A-NS4B-NS5A-NS5B. The structural proteins are C (core) and E1 and E2 (envelope glycoproteins). The function of the p7 product is presently unknown. The NS2 through NS5 regions encode the non-structural proteins [3].

Six major HCV genotypes and about 50 subtypes have been described [4], [5] based on levels of sequence divergence. HCV genotypes have been shown to be distributed over distinct geographical areas and although they share most basic biological features, there seems to be some differences in their susceptibility to interferon (IFN)-based therapies [6], [7]. Genotype 1 is the predominant variant in developed countries and shows the poorest response to therapy. Patients with genotypes 2 and 3 are also common in Europe, although with a lower frequency than genotype 1, and show the best response to IFN therapy.

HCV is mainly transmitted by parenteral routes and differences in their transmission rates can be an important factor to explain the differences in prevalence of a genotype/subtype in different geographic regions [8]–[10]. Needle sharing among intravenous drug users (IDUs) currently represents the most common route of acquisition of HCV in the developed world [11]. Because HCV and human immunodeficiency virus (HIV) share blood-borne transmission routes, HIV/HCV co-infection is relatively frequent, especially in regions such as Spain, where the major proportion of newly diagnosed AIDS patients belong to the IDU category (44.2%) [12].

Like HIV-1, HCV is characterized by high levels of genetic heterogeneity [13], [14] which impact heavily on different aspects such as HCV persistence, susceptibility to treatment, progression of infection, among others [15]–[17]. Although it is well known that recombinant forms of HIV-1 have a relatively high prevalence all over the world [18], there has been limited evidence of HCV recombination between different genotypes/subtypes [19], suggesting that these events are rare *in vivo* and that the resulting recombinants are usually not viable [14], [20], [21]. In the last few years, a few natural intergenotypic recombinants of HCV have been identified (RF1_2k/1b, RF2_1a/1b and RF3_2b/1b) and the crossover points have been mapped to the NS2, NS5B and NS3 regions, respectively [22]–[25]. Very recently, a new natural intergenotypic (2/5) recombinant of HCV has been found whose crossover point is located between genes NS2 and NS3 [26]. All these reports have described HCV recombination between different genotypes/subtypes but, to date, there is only a single case of an HCV intra-subtype recombinant strain, detected by analysis of NS5A sequences from intra-patient populations belonging to six patients undergoing anti-viral therapy [27].

Given the important role that recombination seems to play in the evolution of RNA viruses [28], [29], by creating genetic variation, and the important implications that the production of new pathogenic recombinant strains could have, for example, on the development of vaccines to control RNA viruses, our aim in this study has been to assess the extent and, eventually, the frequency of intragenic recombination on HCV. For this, we have retrospectively analyzed a large data set (over 17700) of HCV sequences from intra-patient viral populations. These sequences were obtained from two separate studies of our group none of which was specifically designed for this objective [10], [78], [79, and unpublished results]. One included only HCV-monoinfected patients and the other HCV/HIV coinfected patients, with a common genome region for both studies (E1-E2 region) and another, the NS5A region, analyzed only in the former study. Both studies included treatment-naïve and patients non-responding to antiviral treatment. We found evidence of intrapatient recombination in HCV sequences from over 10% of the patients thus revealing that recombination in HCV can be a much more common phenomenon than previously recognized. The possibility of this result arising from artifacts during the experimental procedure has been considered by performing an “ad hoc” experiment in which serum samples from two closely related, but clearly differentiated patients were mixed in equal proportions and the resulting mixture was subjected to the same experimental procedure used in the previous analyses. No evidence of artifactual recombination was found.

## Results

Sequences used in this work were derived from two previous studies on HCV genetic variation before and after antiviral treatment. One study included HCV-monoinfected patients whereas the other analyzed HCV/HIV-coinfected individuals. None of these studies was designed with the goal of detecting recombination in HCV. In both cases the E1-E2 region of the HCV genome was analyzed by sequencing viral clones and the former study also included clones from the NS5A region. We obtained sequences from the E1-E2 region from 110 patients and a total of 136 samples (Table 1), since two samples (before and after antiviral treatment) were available for 26 patients, all from the HCV-monoinfection study. For the NS5A region we obtained cloned sequences from 78 patients and a total of 98 samples, since amplification failed for 4 and 1 samples from the pre- and post-treatment groups, respectively.

**Table 1:** Summary of patients, sequence data sets and recombination analysis results.

	Source	Patients	Group	Amplified samples	Average sequence/ sample	SD	Total seqs	Positive cases	Freq.
E1-E2 region	HCV	78	HCV0	77	105.74	21.43	8142	7	0.091
			HCVT	26	106.15	5.24	2680	4	0.154
	HCV-HIV	16	HCV0-0	16	28.69	3.86	433	2	0.125
		17	HCV0-T	17	28.88	7.32	491	3	0.176
	TOTAL	111		136			11746	16	0.118
NS5A region	HCV	78	HCV0	73	63.33	18.51	4623	7	0.096
			HCVT	25	53.72	18.00	1343	2	0.080
	TOTAL	78		98			5966	9	0.091

The “source” column represents the study (HCV-monoinfection, HCV/HIV-coinfection) in which samples were obtained originally. For the HCV-monoinfection study, samples were obtained from 78 patients and the numbers indicated in the “amplified samples” column yielded successful amplificates before (HCV0) and after (HCVT) antiviral treatment. For the HCV/HIV study, the two groups correspond to patients without any treatment (HCV0-0) or having been treated for HIV infection (HCV0-T). SD indicates standard deviation of the number of sequences obtained from each sample and “Freq.” denotes the frequency of samples in which at least one recombination event has been detected.

The average number of clones sequenced for each region, group and patient is described in Table 1. For the E1-E2 region, 11746 cloned sequences were obtained and average number of sequenced clones per sample for the treatment-naïve mono-infected group (HCV 0) was 105.74±21.43 whereas the average for HCV treated mono-infected patients who did not respond to a combined antiviral treatment with IFN-α plus ribavirin (HCV T) was 106.15±5.24. In the case of HCV/HIV coinfected patients, an average of 28.69±3.86 and 28.88±7.32 E1-E2 viral sequences were obtained for treatment naïve (HCV 0-0) and HIV treated patients (HCV 0-T), respectively. The data set for the NS5A region comprised 5966 clonal sequences. The average number of NS5A clones sequenced per sample for the non-treated group (HCV 0) was 63.33±18.51 and 53.72±18.00 for the antiviral treated group (HCV T).

**Figure 1:**
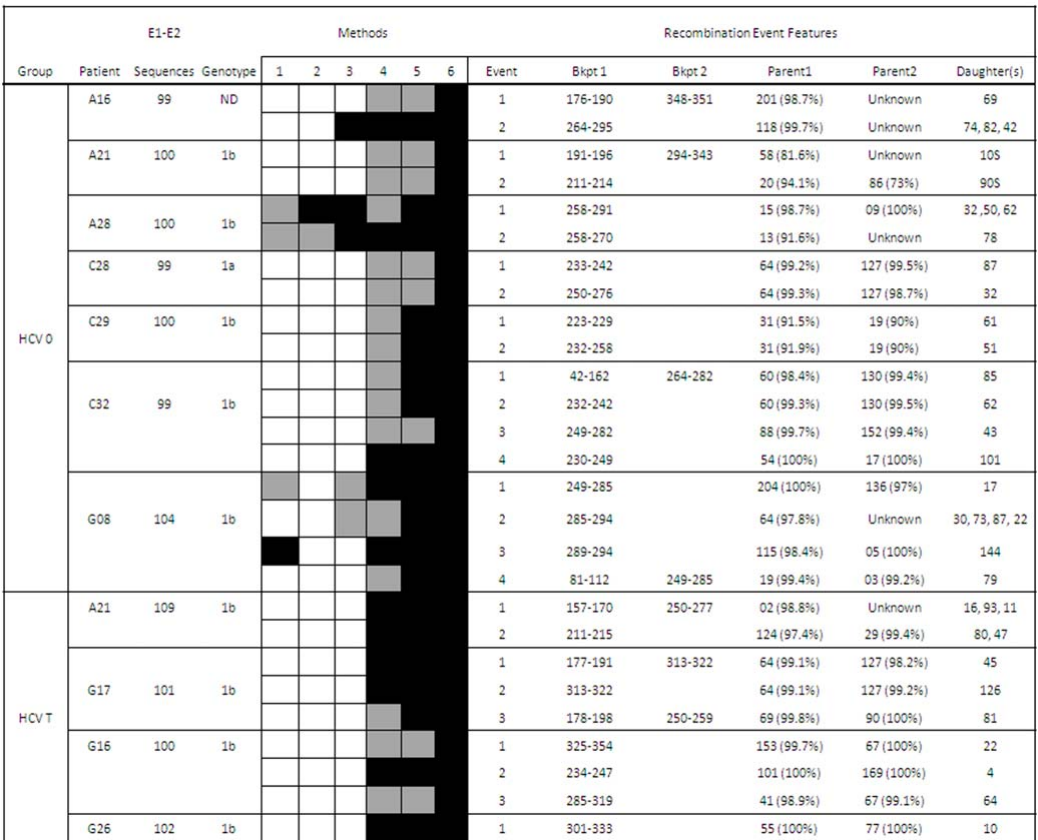
Summary of positive recombination results in the E1-E2 region (HCV-monoinfected patients). The columns represent: i) patient group before (HCV 0) or after (HCV T) antiviral treatment; ii) patient code; iii) amplified sequences for this patient and region; iv) HCV genotype; v) p-values for the different recombination detection methods implemented in RDP 3.0 (1 = RDP, 2 = Geneconv, 3 = Bootscan, 4 = Maxchi, 5 = Chimera, and 6 = Siscan) using the following color coding: non-significant p-values (white filling), p<0.05 (grey) and p<0.01 (black); vi) recombination event number; vii) Bkpt 1, location range for breakpoint 1; viii) Bkpt 2, location range for breakpoint 2; ix) parent1 sequence (% similarity to daughter sequence); x) parent2 sequences(% similarity to daughter sequence); and xi) daughter sequence(s). Only events detected as significant by at least three methods are shown.

Putative recombination events between the viral strains infecting the same patient were found in 20 of the 111 patients studied (18.01%). These intragenic recombination events were detected in 25 of the 234 independent samples analyzed (10.7%). The detected recombination events belonged to HCV samples from all the infection groups described and the two genomic regions analyzed (E1-E2 and NS5A), as well as to different HCV subtypes (1a, 1b and 3a). Five events were detected among the E1-E2 sequences derived from HCV/HIV coinfected patients, 11 corresponded to events in the E1-E2 region from HCV-monoinfected patients, and the remaining 9 were detected in the NS5A region. No differences between the recombination frequencies from the two genomic regions analyzed were found neither for treatment-naïve (Mann-Whitney test: z = −0.104, p-value = 0.917) nor for interferon plus ribavirin treated groups (Mann-Whitney test: z = −0.810, p-value = 0.418). Figures 1–4 show in detail the results of recombination analyses only for samples in which the presence of putatively significant recombination events was detected. Intragenic recombination analyses were performed for each locus independently using 6 different methods for the detection of recombination implemented in the program RDP 3.0. We considered as significant recombination events only those for which the corrected probability for simultaneous inference of the event was lower than 0.05 and were significantly detected by at least 3 of the 6 methods used.

**Figure 2 pone-0003239-g002:**
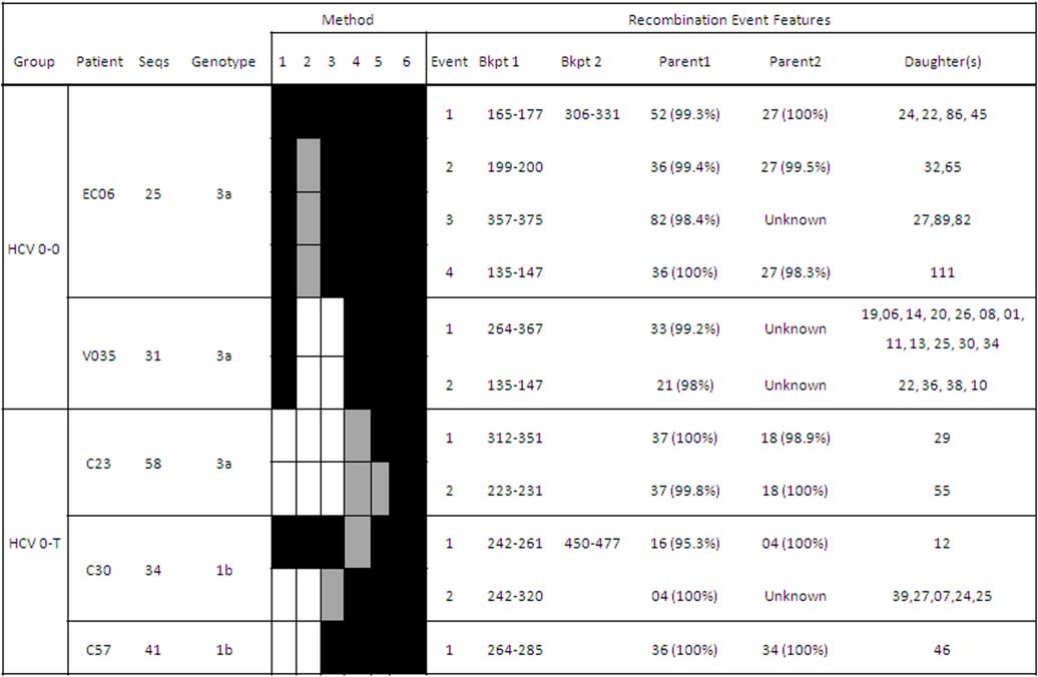
Summary of positive recombination results in the E1-E2 region (HCV-HIV coinfected patients). See further details in legend to Figure 1.

**Figure 3 pone-0003239-g003:**
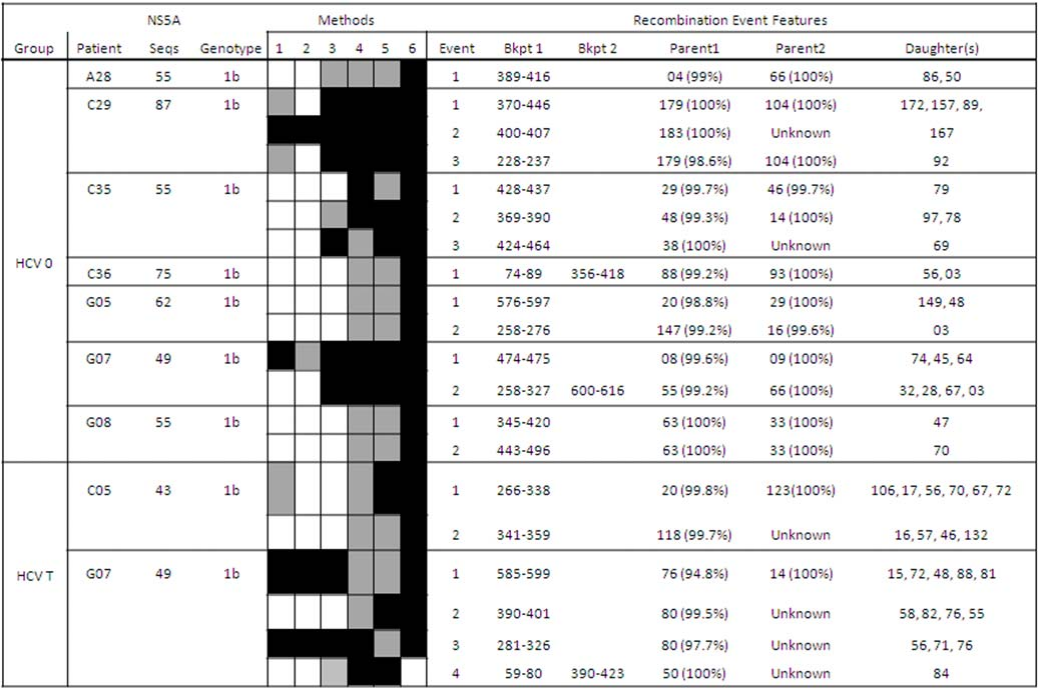
Summary of positive recombination results in the NS5A region. See further details in legend to Figure 1.

**Figure 4:**
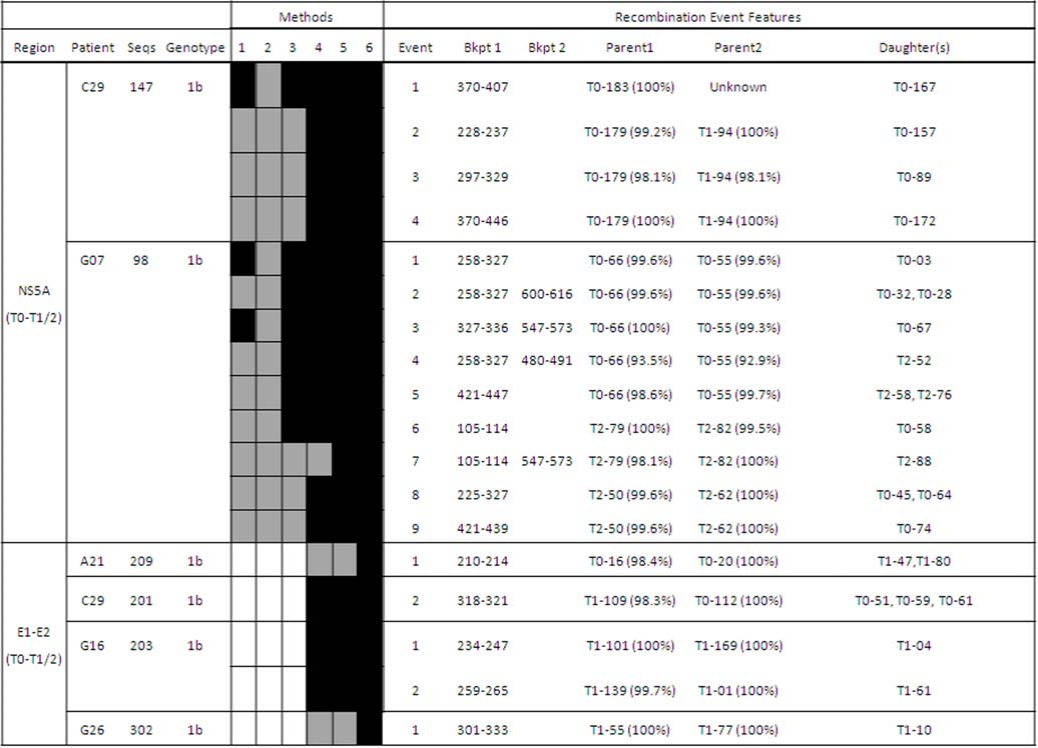
Summary of the joint analysis of two time-point samples from the same patients resulting in positive detection of recombination. See further details in legend to Figure 1.

A large proportion (84%, 21/25) of the samples in which recombination was detected included more than one recombination event (Figures 1–4). Each recombination event detected in a sample was described according to the following features: i) average p-value for the event and analysis method, ii) most likely parental and daughter sequences as well as similarity among them, and iii) most likely limits for the location of breakpoint(s) in the sequenced fragment.

We found 46 single recombination events (17 in the NS5A and 29 in the E1-E2 region) and 12 double recombination events (3 in the NS5A and 9 in the E1-E2 region) in the 25 alignments where recombination was detected. A single recombination event was defined by a single, significant breakpoint detected in the daughter sequence following the described methodology, while a double event was defined as two significant breakpoints identified in the same daughter sequence, delimiting the extension of the recombinant fragment. When one of the parental sequences implicated in an event could not be inferred within the sequence alignment, it was denoted as “unknown parental”.

Among the methods used, those detecting a larger number of significant events were Siscan, Chimera, and Maxchi, followed by Genconv and the phylogenetic methods, Bootscan and RDP, which detected very few events.

Maximum likelihood phylogenetic trees were constructed for the two regions delimited for each single breakpoint event identified. For the double events detected, three maximum likelihood phylogenetic trees were constructed; one was derived from the alignment for the region involved in the recombinant segment (delimited by the two breakpoints detected) and the others from the two resulting flanking segments. Two different tests, Shimodaira–Hasegawa (SH) and Expected Likelihood Weight (ELW), were applied to the two or three resulting topologies for each single or double event, respectively, to further verify the results obtained with RDP3. Tests for double events were made between the recombinant segment topology and each of the two topologies derived from the flanking segments independently. These tests resulted in significant differences between both topologies in all single and double event cases (detailed results of the tests are available in the Supporting Information Table S2).

### Recombination in the E1-E2 Region

**Figure 5:**
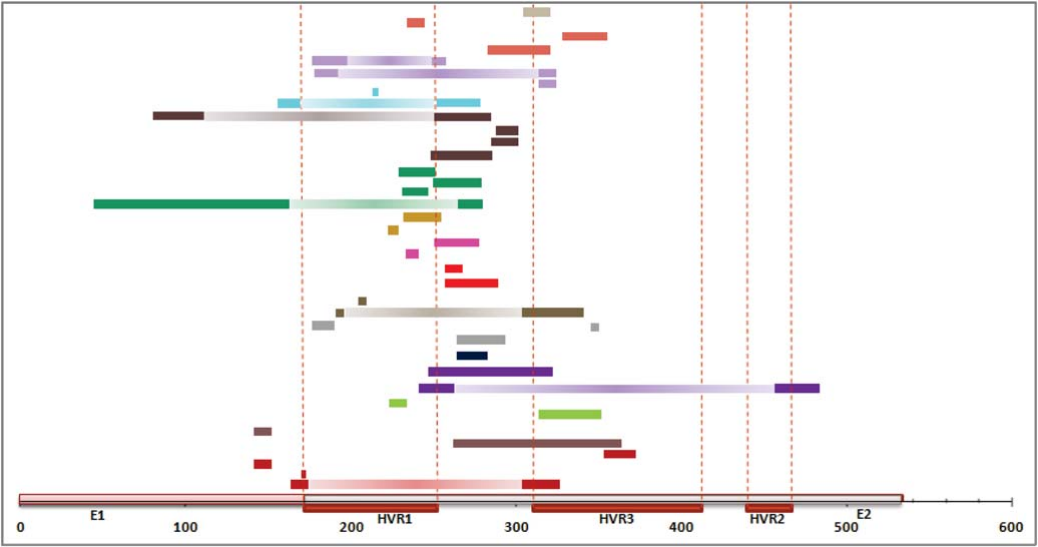
Location of recombination breakpoints detected in the E1E2region.

Statistically significant recombination events were detected by at least three methods in 11.8% (16/136) of the amplified samples in the E1-E2 region. The total number of events identified in these recombinant samples was 38, corresponding to 29 single and 9 double events, belonging to 15 patients from all the studied groups. The breakpoints detected were located mainly in the segments flanking the HVR1 (Figure 5). No differences in the distribution of breakpoints were found between the different studied groups, viral subtypes or treatment response. The frequency of double recombination events detected in this region was 23.7% (9/38) and the average length of the derived recombinant fragments was 147.5 nt (ranging from 66.5 to 212 nt), representing between 27.2% and 31.2% of the total region sequenced (543 nt for HCV/HIV co-infected and 472 nt for HCV single-infected patients). In 8 of the 9 double recombination event cases the derived recombinant regions comprised a large portion of the complete HVR1, while in the remaining case the recombinant fragment involved the entire HVR3 region (Figure 5).

In the analysis of HCV-monoinfected samples (103 E1-E2 amplified samples from 78 patients), the frequency of significant recombination cases was 9.1% (7/77) for treatment-naïve patients (HCV 0) and 15.4% (4/26) for IFN-α plus ribavirin treated patients (HCV T, Table 1). All samples in which recombination events were detected were derived from patients infected with genotype 1b of HCV, including both responders and non-responders to antiviral treatment, with the only exception of a sample from an untreated patient infected with genotype 1a. The frequencies of samples with detected recombination events among coinfected patients were 12.5% (2/16) and 17.64% (3/17) for treatment-naïve (HCV 0-0) and HAART-treated (HCV 0-T) patients, respectively (Table 1). These five samples were obtained from individuals infected with genotype 3a (3 patients) and genotype 1b (2 patients) of HCV. No significant differences were found in the frequencies of recombination-positive samples from naïve and treated groups for HCV mono-infected and HCV/HIV co-infected patients (Mann-Whitney test for single infected patients: z = −0.894 p-value = 0.371; and z = −0.406 and p-value = 0.685, for co-infected patients), although a larger proportion of recombination events was detected among samples from treated patients both in single-infected and in co-infected individuals. Similarly, no differences were found between HCV/HIV co-infected and HCV single infected, treatment-naïve groups (z = −0.417, p-value = 0.676). Complete Mann-Whitney tests results are available in Table S3 of the Supporting Information.

**Figure 6:**
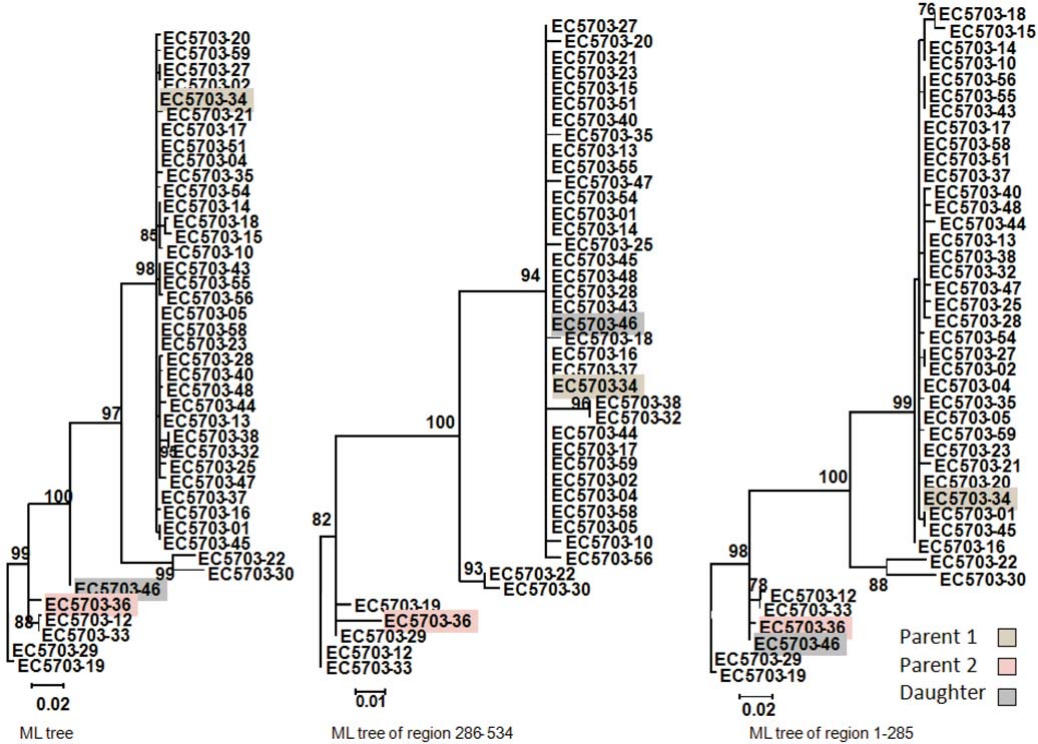
Example of recombination detection in the E1E2 region (EC5703 sample). The phylogenetic tree on the left corresponds to the analysis of the complete region whereas the other two are derived from the two regions defined by the detected recombination event.

**Figure 7:**
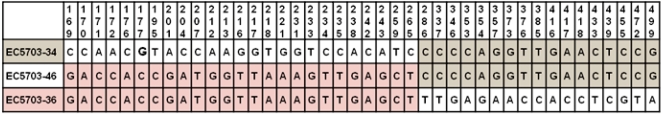
Example of recombination detection in the E1E2 region (EC5703 sample). Variable nucleotide positions in the daughter (EC5703-46) and two parental sequences (EC5703-34 and EC5703-36).

Figure 6 shows an example of a recombination event detected by phylogenetic analysis in the E1-E2 region reflected in the incongruence (reciprocal tests with SH and ELW were highly significant, see Supplementary material) between the maximum likelihood trees derived from the two regions (delimited by nucleotides 1–264 and 265–534, respectively) defined by the breakpoints assigned to the recombination event from sample EC5703. Variable positions in the alignment of the viral sequences involved in this recombination event are shown in Figure 7, marking the recombinant parental sequences involved in the detected recombination event.

### E1-E2 Region. Analysis of Two Time-Point Samples

Two samples were available for 24 HCV-monoinfected patients who did not respond to antiviral treatment. One sample was taken before the onset of treatment (T0 sample) and the second one was obtained when it was discontinued, 6 or 12 months later (T1 sample). For these cases an additional analysis was performed by simultaneously considering all the sequences obtained from both samples. We detected significant recombination events in 4 patients, C29, A21, G16 and G26.

One recombination event was detected in the joint analysis of the two samples from patient C29 (C29T0-T1).The parental sequences from this event were derived one from the T0 sample (before treatment) and the other from the T1 sample (taken 6 months later), while the daughter sequences were detected only in the T0 sample. The same event, involving the same daughter sequences, was also detected in the analysis of sequences obtained only from the T0 sample, although in this case only one parental sequence was identified while the other was described as “unknown parental” in the analysis (Figure 1).

Similarly, in the joint analysis of the two samples from patient A21, we detected one significant recombination event whose corresponding breakpoint was located between positions 210 and 214. This event was originated by two parental sequences from the T0 sample while the resulting daughter sequences from such event (A21_T1-47 and A21_T1-80) corresponded to sequences from the sample obtained after six months of unsuccessful HCV antiviral treatment (Figure 4). The same event was detected in the analysis of sequences derived only from the T1 sample but, obviously, the identified parental sequences belonged also to this sample. However, when the parental similarities for both events (the one detected only with T1 sequences and the other joining T0 and T1 sequences) were compared, the similarity between daughter and the corresponding parental sequences for both cases were lower in the T1 sample (97.4 and 99.4% for the two putative parental sequences) than in the joint T0-T1 analysis (98.4 and 100%). In consequence, T1 daughter sequences derived from this recombination event had been more likely generated by parental sequences from the T0 sample than from those in the T1 one. This would imply that the recombination event occurred at the earlier time point and the resulting daughter sequences were able to persist in the viral population six months later and under antiviral treatment. Finally, in the case of patients G16 and G26, all significant events detected in the joint analysis involved sequences from the same time-point and were also detected in the analysis of only this sample.

### Recombination in the NS5A Region

**Figure 8:**
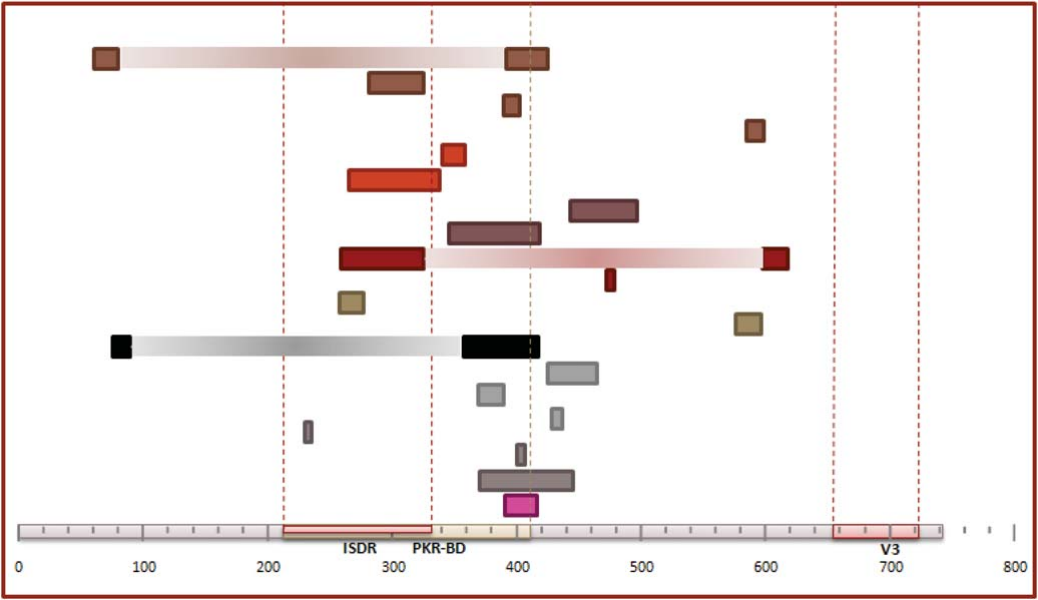
Location of recombination breakpoints detected in the NS5a region.

**Figure 9:**
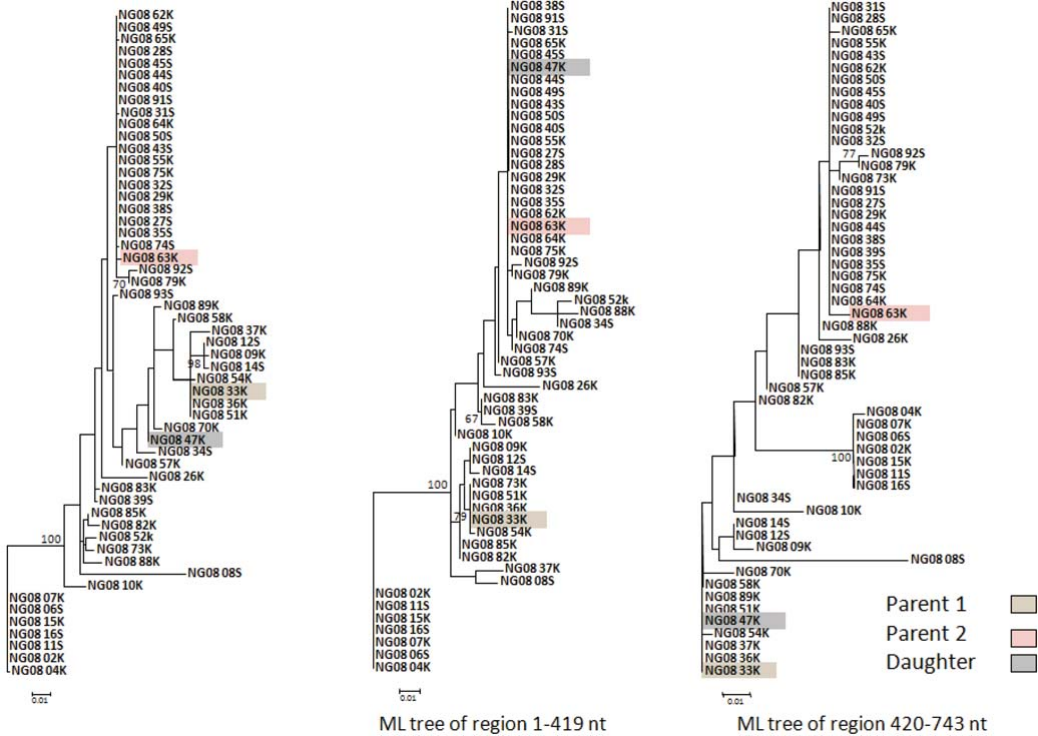
Example of recombination detection in the NS5a region (NG08T0 sample). The phylogenetic tree on the left corresponds to the analysis of the complete region whereas the other two are derived from the two regions defined by the detected recombination event.

**Figure 10:**
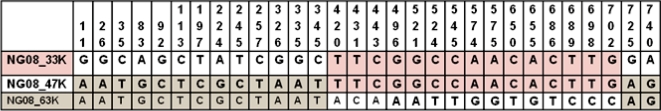
Example of recombination detection in the NS5a region (NG08T0 sample). Variable nucleotide positions in the daughter (NG08-47K) and two parental sequences (NG08-33K and NG08-63K).

Significant events of recombination were detected by at least three methods in 9.18% (9/98) of the amplified samples for the NS5A region. The total number of recombination events detected in these samples was 20, with 17 single and 3 double events, and they were identified in samples from 8 patients, all infected with HCV genotype 1b, including treatment-naïve and non-responder patients. The breakpoint(s) detected for each event were located mainly in a segment flanking the PKR-BD (protein-kinase binding domain) and within the ISDR (interferon sensitivity-determining region) regions (Figure 8). The frequency of double events detected in this region was 15% (3/20) and the average recombinant fragment length was 315 nt (ranging from 305 to 337 nt) corresponding to the 42.4% of the total sequenced region (743 nt). In 2 of the 3 double event cases, the recombinant region comprised the complete PKR-BD region, including also the ISDR, while in the other case the recombinant region involved only the non ISDR fragment of PKR-BD region (Figure 8). No differences were observed in the distribution of breakpoints between treated and non-treated samples or between treatment responses. Similar proportions of recombination events were detected in samples from treatment-naïve patients (9.6%, 7/73) than from IFN-α plus ribavirin treated patients (8.3%, 2/24) and no significant differences due to the HCV treatment were found (z = −0.236 p-value = 0.813). Figure 9 presents an example of recombination event detected in the NS5A region from the T0 sample of patient G08. In this case, the event corresponded to a single recombination breakpoint located between positions 345 and 420 of this fragment. Maximum likelihood trees obtained for each region using the GTR model of evolution showed a clear phylogenetic incongruence between the two derived region trees (Table S2 and File S1 in Supplementary material). Variable positions in the alignment of the viral sequences involved in this recombination event are shown in Figure 10.

### NS5A Region: Analysis of Two Time-Points Samples

Two samples, one taken before (T0) and the other after unsuccessful antiviral treatment (6 or 12 months later, T1 or T2), were available for 24 HCV-monoinfected patients. The joint analysis of sequences from the two samples of each patient resulted in the detection of recombination events in only two cases (8.33%), for patients C29 and G07.

The joint analysis of samples from patient C29 revealed several recombination events that involved parental sequences from samples T0 and T1 and daughter sequences only from the T0 sample. All these events were also detected in the analysis of the T0 sample but, again, with presumed parental sequences from this sample time point.

For patient G07, different recombination events in the joint analysis of sequences from samples T0 and T2 were found. Two of these events were particularly interesting because they involved daughter sequences from the T2 sample with inferred parental sequences from the T0 sample which was obtained one year earlier. These parental sequences (T0-66 and T0-55) were involved in other recombination events detected in the T0 sample and the T2 resulting daughters (except T2-52, only detected in the joint analysis), T2-58 and T2-76 were detected in the T2 sample too, but parental inferred sequences for these events had lower similarity or were unknown (see Figures 3 and 4). Similarly to the previously commented case of E1-E2 sequences from two time-points samples of patient A21, the T2 daughter sequences obtained from this event were more likely generated by parental sequences from the T0 sample. Again, this indicates that the recombination event most likely took place at T0 and the resulting daughter sequences were able to persist for one year under HCV treatment.

### Joint Analysis of Recombination in the E1-E2 and NS5A Regions

We obtained sequences for the E1-E2 and NS5A regions from 73 patients. These were all from the HCV monoinfected, non-treated group (HCV 0). Given the observed frequency of recombination events in the two analyzed regions, we calculated the expected probability of detecting recombination events simultaneously in both genome regions for the same patient. The frequency of samples with significant recombination events detected was 9.1% (7/77) and 9.6% (7/73), for the E1-E2 and NS5A regions, respectively (Table 1). Hence, the expected frequency of obtaining a patient with recombination events detected in the two genomic regions if these events were independent was 0.87%, whereas the observed frequency was 4.1% (3/73), 4.45 times higher than the expected value. Fisher's exact probability test resulted in a significant value with p = 0.03.

We have also compared the frequencies of the recombinant sequences with those of the other sequence clones. A summary of these results is shown in Table 2. While the frequencies of detected recombinant sequences varied widely, from a minimum of 0.003 to a maximum of 0.516, none of the recombinant sequences was present in more than 2 copies in the corresponding population, which corresponded to frequencies from 0.001 up to 0.074. Nevertheless, these figures must be compared with those corresponding to the frequency of the most common variant in the corresponding population, which varied between 0.030 and 0.20. In the joint analyses of two samples from the same patient in which recombinant sequences were detected, the highest frequency of a recombinant haplotype was 0.010. The most common sequence in this patient was only three times larger (0.031) but the largest number of identical sequences (111) and the highest haplotype frequency (0.547) were found in this group.

**Table 2:** Summary of frequency analysis of recombinant sequences in the different data sets where intrapatient recombination of HCV was detected.

Group	Patient	Total Seqs.	Diff. Haplotypes	Numb. of recomb	Freq. recomb.	Recombinants		Non-recombinants	
						Seqs.	Freq.	Seqs.	Freq.
NS5a region HCV 0	A28	55	53	2	0.036	1	0.018	2	0.036
	C29	87	32	5	0.057	1	0.011	16	0.184
	C35	55	53	4	0.073	1	0.018	2	0.036
	C36	75	67	2	0.027	1	0.013	3	0.040
	G05	62	50	3	0.048	2	0.032	3	0.048
	G07	49	45	7	0.143	1	0.020	2	0.041
	G08	55	32	2	0.036	1	0.018	17	0.309
HCV T	C05T1	43	39	10	0.233	1	0.023	2	0.047
	G07T2	49	45	13	0.265	1	0.020	2	0.041
E1-E2 region									
HCV 0	A16	99	92	4	0.040	1	0.010	4	0.040
	A21	100	72	2	0.020	1	0.010	14	0.140
	A28	100	87	4	0.040	1	0.010	3	0.030
	C28	99	77	2	0.020	1	0.010	3	0.030
	C29	100	37	2	0.020	1	0.010	20	0.200
	C32	99	80	4	0.040	1	0.010	5	0.051
	G08	104	93	6	0.058	1	0.010	4	0.038
HCV T	A21	108	84	5	0.046	2	0.019	6	0.056
	G17	100	81	3	0.030	1	0.010	6	0.060
	G16	100	79	3	0.030	1	0.010	6	0.060
	G26	102	58	1	0.010	1	0.010	20	0.196
HCV 0-0	C06	25	21	10	0.400	2	0.080	3	0.120
	V035	31	26	16	0.516	2	0.065	2	0.065
HCV 0-T	C23	29	29	2	0.069	1	0.034	1	0.034
	C30	27	19	6	0.222	2	0.074	3	0.111
	C57	41	31	1	0.024	1	0.024	8	0.195
Two joint samples									
NS5A	C29T0-T1	147	59	4	0.027	1	0.007	34	0.231
	G07T0-T2	98	89	13	0.133	1	0.010	3	0.031
E1-E2	A21T0-T1	209	156	2	0.010	2	0.010	14	0.067
	C29T0-T1	200	71	3	0.015	1	0.005	20	0.100
	G16T0-T1	203	82	2	0.010	1	0.005	111	0.547
	G26T0-T1-T2	302	31	1	0.003	1	0.003	85	0.281

For each patient and sample(s) considered the table reports the total number of sequences analyzed, the number of different haplotypes, the total number of recombinant sequences and their frequency in the sample. Additional information is given on the absolute and relative frequencies of the most frequent haplotype among recombinant and non-recombinant sequences.

### Experimental Analysis of Artifactual Recombination

In order to evaluate the level and extent of artifactual generation of recombinant sequences during the reverse transcription and PCR amplification, we performed an additional experiment using the same conditions previously described. The only difference was the starting sample used which, in this case, was a mixture of sera derived from two patients. These were chosen to maximize the possibility of detection of recombinant sequences, which requires clearly differentiated viral populations, while simultaneously maximizing the possibility of contiguous pairing enabling RNA polymerase shifting from one template to another while keeping the same position and reading-frame. This was achieved by selecting two patients HCV-1b infected from a common source who had within-patient nucleotide diversities of 0.0046 and 0.0007, respectively, with a net nucleotide differentiation of 0.0247, as estimated from a previous analysis of 10 clones from each sample (unpublished results).

To ensure equimolar amounts of viral RNAs from both samples in the final mixture, we set up several mixtures with varying amounts of each sample that were subjected to the same RT and PCR-amplification procedures for the E1-E2 region described above. The resulting amplificates were directly sequenced and one mixture with equal peak heights in the automated sequencer electrophoregram in the polymorphic positions was chosen for further analysis. We cloned and sequenced PCR products from the selected mixture as described, obtaining 142 sequences. These were analyzed using the 6 methods implemented in RDP as described above and none of them detected any putative recombination event.

## Discussion

Several studies have reported recombination in different Flaviviruses [30]–[33], but until recently no evidence for recombination in natural populations of HCV had been found. Since the first identification of an intergenotypic (2k/1b) HCV recombinant in St. Petersburg [22], several intergenotypic and intragenotypic HCV recombinant strains have been identified [23]–[26], [34], [35] therefore incorporating recombination as a mechanism generating genetic variation in HCV. More importantly, the identification of these recombinant strains demonstrates that HCV is capable of successfully completing all the stages (simultaneous infection of the same cell, simultaneous replication of both viral genomes, template shift by the viral RNA polymerase while keeping the correct reading frame, encapsidation and release of the recombinant genomes) in the process. The resulting products will then be subjected to the same population processes governing the maintenance, expansion or disappearance of new variants in a heterogeneous viral population.

All these reports have focused on HCV recombination between different genotypes/subtypes, but to date, there is only one single case of putative HCV intra-subtype recombinant strain, detected by the analysis of NS5A sequences from six intra-patient populations undergoing antiviral therapy [27]. In the present study we have identified a high frequency of intrasubtype recombination events (18.01% of the analyzed patients 20/111) analyzing a large data set of HCV sequences from intra-patient viral populations obtained from patients belonging to different groups: HCV-monoinfected patients, naïve and non-responding to antiviral treatment, and HCV/HIV-coinfected patients, treatment-naïve and under HAART. The relevance of recombination in HCV for its long-term evolution and its incidence in different aspects of HCV infection have not been explored yet, but these findings support a potentially significant role for recombination in the evolution of HCV by creating genetic variation through the reshuffling of independently arisen variants.

Although these studies have firmly established the possibility of recombination in HCV, as in other Flaviviruses, no general mechanism has been proposed yet (but see below), and despite extensive analysis of genetic variation in HCV there has been only one report of recombination between strains from the same subtype. Hence, it seems adequate to start discussing our findings in terms of them being real or an artefact and, since we naturally accept the first option, why it has been so difficult to detect.

The first question is how to discard the possibility that the detected recombination events were false positives, resulting from PCR-mediated recombination, especially since some experiments have failed to experimentally induce and detect recombination in HCV [36]. We have performed one further such experiment, also with negative results. This is no proof of absence of artifacts in our experimental results, but they clearly indicate that, if present, recombinant sequences arising from the experimental procedures cannot account for the reported results. This conclusion is based on the following arguments, derived from considering different possible estimates of the artifactual recombination rate when no such event has been observed. If no false recombination event is observed among 143 clones then the probability of any such event must be lower than 1/143 (0.0075). With this upper limit, which would be our worst case scenario, under a Poisson distribution we would have expected to observe no false recombination events in 138.5 of the 234 independent samples analyzed with an average size of 75 sequences each. The actual number of samples with no recombinant sequences detected was 210; hence the presumed rate of artifactual recombination must be lower than 0.0075. If we had based our estimate of artifactual recombination rate on the number of negative cases (210), the inferred rate using again a Poisson distribution would be 0.0014. But with this rate it is not possible to account for the observation of 21 cases with two or more recombination events (Figures 1–3) nor of up to 9 events when sequences from the same patient taken at two different times were combined in a single analysis (Figure 4). Finally, should we consider the 58 events observed among 17712 sequences analyzed to be artifacts then we would expect 183 and 45 samples with none and one recombination event, respectively, while we obtained 210 and 3 cases. The discrepancy for cases with 2 or more events is even larger (5, 0.5 and 0, for 2, 3 and 4 events, while 13, 4 and 4 have been observed, respectively). In summary, the observed number and distribution of recombination events cannot be explained by artifacts during the experiments. Additional arguments for this conclusion are discussed next.

There are two points in our experimental procedure that have been instrumental in obtaining the reported results and building our confidence in that they are not artifacts. First, instead of analyzing the full length master sequence of the viral distribution in each sample, we concentrated our efforts in sequencing a large number of clones in each of two genome regions. These were chosen on the basis of their biological relevance and not on the location of breakpoints identified in previous reports of recombination in HCV [23]–[26], [34], [35]. Secondly, we minimized the chances of detecting false, artifactual recombination by using long extension times [37] and a proofreading DNA polymerase (Pfu) [38] in our PCRs. Additional support for the actual occurrence of recombination events within HCV infected patients is provided by the following points: i) breakpoints or recombinant regions implicated in the recombinant events are not distributed at random along the analyzed genome regions (Figures 5 and 8). On the contrary, the same breakpoints have been detected in HCV samples from different patients, indicating the presence of recombination hotspots, and entire biologically relevant regions are comprised within the recombinant fragments detected. ii) We have found evolution of the daughter sequences derived from a recombination event, with similarity to the parental sequences lower than the expected 100% if the recombination event was PCR-mediated. In addition, all recombination generated sequences preserve the polyprotein reading frame, which is not expected in PCR-mediated recombination where selection cannot purge deleterious mutants. iii) We have detected several recombination events in which the most likely parental sequences belonged to a previous time sample. Through the joint analysis of sequences from the two sample time points from a single patient, we have identified recombinant daughter sequences resulting from the cross-over between sequences sampled six or twelve months before under HCV treatment (see Figure 4, results for A21_T0-T1 and G07_T0-T2). Parental and recombinant sequences were amplified and sequenced independently from the two different samples. Hence, these events probably occurred in the period between the two samples, and therefore the recombinant sequences were able to persist in the viral population under treatment and immunological selection pressures. iv) Finally, we have found patients with evidence for recombination events in the two genome regions analyzed 4.3 times more often than expected from the assumption of complete independence between both events. In PCR-mediated recombination we would expect a similar frequency of independent recombination events in both regions, but the joint expectation would be their product. A substantially larger value such as that observed might indicate the existence of some features, probably in the viral RNA-dependent RNA-polymerase (protein NS5B in HCV) that might either facilitate or prevent recombination, thus leading to the observed discrepancy.

Intergenotype recombination in HCV has been related to homologous recombination during minus-strand synthesis via template switching [23], although this proposal relies on the presence of two hairpin structures in the vicinity of the inferred breakpoint of recombination. Recombination breakpoints for the E1-E2 region were mainly located in the conserved region between hypervariable regions 1 (HVR1) and 3 (HVR3), and the recombinant fragments from the double recombinant sequences detected spanned the entire HVR1 or HVR3 regions. For the NS5A region, breakpoints were concentrated mainly at the end of the ISDR and the PKR-BD, and double recombinant fragments comprised the entire ISDR or PKR-BD regions. This distribution of breakpoints could be explained by the operation in HCV, like in other DNA or RNA viruses, of an intermolecular homologous replicative recombination system. This mechanism is associated with extensive nucleotide sequence identity between the two parental genomes around the cross-over site and copy choice or template switching during the replication process, that involves detachment from a template of the polymerase complex with a nascent product, and continuation of the copying process at the same position of another template molecule.

This is the first study where recombination in HCV has been detected at the intrapatient intragenic level (the lowest possible level of diversity) by analyzing a large number of HCV sequences (17712) and samples (234) from 111 patients assigned to different clinical groups. The detection of recombinant strains in 18% of the HCV-infected patients studied implies that recombination events between the viral strains infecting the same patient may be relatively frequent, and still more if we consider that this might be an underestimate of the true frequency of HCV recombination because of the difficulty in detecting recombination events if they occur between genetically very similar variants of the same subtype or in conserved genome regions.

The frequent detection of recombination events in all patient groups described makes the capability of HCV to produce recombinant forms not only relatively frequent but also effective and, depending on the recombinant strains produced, it might be selectively advantageous. However, we did not find any evidence for an increased frequency of recombinant sequences which might be explained by their presumed selective advantage. A more adequate analysis of positive selection on this same set of sequences does not show any indication of a selective advantage of these recombinant sequences (Sentandreu et al., in prep.).

Given the previously reported results, a higher frequency of recombinant HCV strains than actually identified, with only 5 inter-genotypic and 1 intra-genotypic recombinants reported, might be expected. This might be explained by three different factors: Firstly, in recombination events between subtype viral strains, such as those reported here, there is a trade-off between the capability of homologous replicative recombination event to occur, which likely depend on the intrinsic recombination rate of HCV, and the intra-patient viral diversity, because homologous recombination requires a minimum length of sequence identity. Secondly, there is another trade-off between the intra-patient viral diversity and the power for the detection of recombination by the different methods used. Finally, recombination events between different genotypes/subtypes co-infecting the same patient are most probably easier to detect but, on the other hand, they are less likely to occur, given the higher differentiation between strains of different subtypes than those from the same subtype resulting in less likely template switching and, additionally, if a recombination event does happen it will likely generate recombinant sequences less viable than the parental ones. The action of some or even all these factors thus provides an explanation for the low frequency of recombinant HCV sequences reported up to date.

We have detected recombination events in all the genotypes/subtypes analyzed. However, given the direct relationship between intrapatient genetic variability and our ability to detect recombination events, those viral populations with higher rates of intrapatient genetic variation are more likely to be involved in the detection of a recombination event if it ever occurs. This large genetic variation at the intrapatient level is usually associated to long persistent infections and/or to coinfection and superinfection with a strain from the same or a different subtype/genotype [39]–[41]. Furthermore, there is increasing evidence for the presence of compartmentalization of HCV populations within infected patients [42]–[47], which would further facilitate lineage divergence within a patient. Despite some failed attempts to induce superinfection in cell cultures infected with HCV [48], [49] this and previous reports of recombination in HCV clearly demonstrate that this process is not fully blocked, and more research is certainly necessary to establish under what circumstances and in which cellular types is superinfection with HCV more likely to occur.

Our aim in this study has been to detect the presence of intragenic recombination and also to assess the extension and the frequency of these recombination events at the subtype level, analyzing two genome regions, E1-E2 and NS5A, from HCV-infected patients with different clinical and epidemiological backgrounds: mono-infected with HCV or coinfected with HIV, with or without antiviral treatment, and responder or non-responders to this treatment. No significant differences in the frequency of recombination events were detected between the two genomic regions studied (9.1% E1-E2 and 9.6 % NS5A, Table 1) for the treatment-naïve, HCV-monoinfected patients group (HCV 0). On the contrary, large differences were found between these two regions for the group of HCV infected patients who did not respond to interferon plus ribavirin treatment therapy (HCV T). The observed recombination frequencies, 15.4% and 8.0% for the E1-E2 and NS5A regions, respectively, were not statistically significant due to the low sample size of the HCV T group but hinted to a larger genetic variation being generated in the E1-E2 region of non-responder patients. A positive relationship between genetic variability in this region [50]–[54] and in the whole HCV genome [55] with lack of response to antiviral treatment and progression of the infection has been reported, although there are contradictory observations [17], [56]–[58]. In consequence, if this association is real, an increased rate of recombination in this region might contribute to viral resistance to treatment and, consequently, to a higher probability of detection of recombination in non-responder patients. To date, the sensitivity of recombinant forms of HCV to pegylated interferon-based therapy is still unknown, but recombinant forms for HIV do not have the same sensitivity to anti-retroviral therapy than wild type HIV-1 clade B isolates [59]. Furthermore, it has been recently suggested [60] that recombination plays an important role in the evolution of drug resistance in HIV-1 under various realistic scenarios. No significant differences were found in the frequency of recombination events in the E1-E2 region between the two treatment groups in HIV coinfected patients. Nevertheless, a higher proportion of cases of recombination were detected in the HAART treated group (12.5% for HCV 0-0 and 17.6% for HCV 0-T). This might be related to an increase in the selection pressure due to the decrease in HIV load and the restoration of the immune system in these patients.

There are also reports correlating the degree of variability of the ISDR and responsiveness to interferon treatment [61]–[63], again without a complete consensus. However, the association in this case is an opposite one to that found in the E1-E2 region. Departure from a canonical sequence at ISDR has been associated to decreased response to interferon treatment, mainly in Japanese populations [64]–[66], but opposite results have been obtained for European and American ones [67]–[74]. Nevertheless, recent meta-analyses of these reports have provided further support for this relationship [75]–[77]. Hence, the reversed relationship between genetic variability (departure from the canonical sequence) at ISDR and response to interferon treatment might be counterbalanced by recombination, which would allow the maintenance of the canonical sequence at ISDR while maintaining high levels of variation at other genome locations.

Given the biological relevance described about the regions involved in the recombinant fragments, and the distribution of the recombinant cross-over points, it is clearly that the reported intragenic recombinant exploratory activity producing new genomic combinations could play an important role in the HCV evolution with significant consequences for treatment efficiency and the development of vaccines.

Given the obtained results with a high frequency of HCV intragenic recombinant detected strains from patients belonging to the different described groups and the biological relevance related with the regions involved in this recombinant events, we conclude that, recombination must be considered as a potentially important mechanism generating genetic variation in HCV with serious implications in the vaccine and drug treatment optimal development and the response to antiviral therapy.

## Materials and Methods

### Patients and Samples

136 serum samples from 111 HCV-infected patients were analyzed in this study. Patients belonged to two different groups: (i) infected only with HCV, either treated with IFN-α plus ribavirin (denoted HCV T) or treatment naïve (HCV 0), and (ii) HCV-HIV co-infected patients with (HCV 0-T) and without (HCV 0-0) highly active antiretroviral treatment (HAART) against HIV. Samples from the former group were included in a molecular epidemiology study of HCV in the Comunidad Valenciana and have been described in detail elsewhere [10], [78], [79]. Samples from the second group were obtained from the Hospital General de Valencia (Valencia, Spain) and informed, written consent was obtained from all the patients. Both studies were approved by the corresponding ethics committees of the institutions involved. Treatment response for all HCV treated patients is shown in Table S1.1 (Supplementary Material). For non-responder patients from the HCV T group a second serum sample taken after interruption of treatment (6 or 12 months after its start) was available and included in the study.

HCV genotyping was initially performed by a commercial reverse hybridization genotyping assay (Inno-LIPA HCV II; Innogenetics) and later confirmed by nucleotide sequence comparison in the analyzed genome regions. Genotype 1b represented 61.5% of the total HCV-monoinfected patients analyzed, and genotype 1a the remainder 38.5% whereas for HCV/HIV-coinfected patients, the frequency of the different HCV genotypes were 39.4%, 30.3%, 3.0%, 18.1% and 9.1% for genotypes 1a, 1b, 2b, 3a and 4, respectively.

Two HCV genome regions were considered in this study. The first one corresponds to a fragment encompassing the genes encoding envelope glycoproteins E1 and E2. This fragment spanned from positions 1322 to 1793 in the HCV reference genome sequence [GenBank accession no. AF009606, 80] for HCV mono-infected samples (472 nt) and up to position 1855 in HCV-HIV co-infected samples (534 nt). This region will be referred to as “E1–E2 region”. The second region corresponded to a 743 nt fragment from gene NS5A (positions 6742–7484 in the HCV reference genome), referred to as “NS5A region”.

These two genome fragments were chosen because of the biological relevance of the regions included therein. On the one hand, three hypervariable regions are included in the E1-E2 region: HVR1, which seems to be involved in target cell recognition and virus attachment [81]; HVR2, which could be involved in cell surface receptor binding [82]; and HVR3, which could play a role in the process of binding with host cell receptors and virus entry into host cells [83]. On the other hand, two remarkable domains are included in the NS5A region: the V3 domain, seemingly involved in responsiveness to interferon [70], [84], and PKR-BD, which contains the putative interferon sensitivity determining region (ISDR) and seems to be involved in blocking the cellular antiviral response induced by interferon [61], [85]–[87].

### RNA Extraction, cDNA Synthesis and Amplification

Virus RNA was extracted from 200 µl serum by using a High Pure Viral RNA kit (Roche). Reverse transcriptions were performed in a 20 µl volume containing 5 µl eluted RNA, 4 µl 5× RT buffer, 0.5 mM each deoxynucleotide, 0.5 µl random hexamers, 100 U Moloney murine leukaemia virus reverse transcriptase (Promega) and 20 U RNasin ribonuclease inhibitor (Promega). The reactions were incubated at 42°C for 45 min, followed by 3 min at 95°C.

A first PCR round was then carried out in a 100 µl volume containing 10 µl of the reverse transcription product, 0.2 mM each dNTP, 400 nM genomic primer, 400 nM antigenomic primer and 1.25 U *Pfu* DNA polymerase (Promega). For the first set of samples, i.e. those obtained from HCV-monoinfected patients, we used the primers detailed in Table 1 of [88] unless specified otherwise. These primers yielded a 472 nt fragment for the E1–E2 region, while a 543 nt fragment was obtained in this region from HCV/HIV coinfected samples using primers 1-Em1 (5′-CGCATGGCHTGGRAYATGAT), 1-Em2 (5′-GGRATATGATRAATGAAYTGGTC) and 1-Em1 (5′-GGRGTGAARCARTAYACYGG) for genotypes 1a, 1b, 2b and 4, and primers 3-Eg1 (5′-CGWATGGCTTGGGAYATGAT), 3-Eg2 (5′-GGGAYATCATGATGAAYTGGT), 3-Ea1 (5′- GGRGTRAAGCAGTABACRGG) for genotype 3. For region NS5A, subtype 1a: 1-Ng1, 2-Ng1, 1-Ng2, 2-Ng2, 1-Na and 2-Na. For the NS5A region we used primers Ng1 (59-TGGAYGGRGTRCGGYTGCACAGGTA), Ng2 (59-CAGGTACGCTCCRGYRTGCA) and Na (59-CCYTCRAGGGGGGGCAT), which yielded a 743 nt fragment. This region was analyzed only in HCV-monoinfected samples. In all cases, PCRs were performed in an Applied Biosystems 2400 thermal cycler as described [88].

### Cloning and Sequencing of Viral Populations

Amplified DNA products for each region were purified and cloned directly into *Eco*RV-digested pBluescript II SK(+) phagemid (Stratagene). Cloned products for the E1–E2 region or NS5A region were sequenced by using vector-based primers KS and SK (Stratagene).

Sequencing was carried out by using the ABI PRISM BigDye Terminator v3.0 system (Applied Biosystems) on an ABI 3700 automated sequencer. Sequences were verified and both strands were assembled using the STADEN package [89]. HCV sequences obtained in this study have been deposited in GenBank and the corresponding accession numbers are shown on Table S1 in the supplementary material along with the numbers of previously determined sequences [10], [78], [79].

### Sequence and Phylogenetic Analyses

Sequence alignments were obtained with CLUSTALX v1.81 [90]. Optimal models of nucleotide substitution were assessed using the maximum likelihood approach implemented in Modeltest v3.7 [91]. Likelihood scores for each model were estimated in PAUP*4.0b10 [92] and the best model was determined using the Akaike Information Criterion (AIC) [93]. Maximum likelihood phylogenetic trees were obtained with PHYML 2.4.4 [94] using the previously determined models of nucleotide substitution for each genome region and sample, and support for the nodes were evaluated by bootstrapping with 1000 pseudorreplicates.

### Intrapatient Recombination

Putative recombination events in intrapatient sequence alignments of the two genome regions were detected using RDP 3.0b03 [95]. This program implements several methods for the identification of recombinant sequences and recombination breakpoints. We choose six of them: two phylogenetic methods, which infer recombination when different parts of the genome result in discordant topologies, RDP [96]; and Bootscanning [97]; and four nucleotide substitution methods, which examine the sequences either for a significant clustering of substitutions or for a fit to an expected statistical distribution: Maxchi [98], Chimaera [98], GeneConv [99] and Sis-scan [100].

We only considered recombination events that were identified by at least three methods. Common settings for all methods were to consider sequences as linear, to require phylogenetic evidence, to polish breakpoints and to check alignment consistency. Statistical significance was set at the P<0.05 level, after considering Bonferroni correction for multiple comparisons as implemented in RDP. Consensus daughter sequences and breakpoints were determined whenever possible.

In order to test the phylogenetic congruence of the two ML trees derived from each of the segments identified by the recombination breakpoints reported, we used TreePuzzle v.5.2 [101] to compare both phylogenetic trees using the SH [102] and the ELW tests [103].

## Supporting Information

Table S1 Detailed information on patients, samples, sequences and accession numbers used in this research
(0.07 MB XLS)

Table S2 Summary of SH and ELW tests for alternative topologies derived from the recombination events detected
(0.19 MB PDF)

Table S3 Summary of Mann-Whitney tests for differences in recombination frequency between clinical groups considered in this study
(0.09 MB DOC)

File S1 Zip-compressed file with all the maximum likelihood trees used in the analyses
(0.13 MB ZIP)
