# Supplementary material for: Evidence of Recombination in Intrapatient Populations of Hepatitis C Virus
Source: PLoS One. 2008 Sep 18;3(9):e3239. doi: 10.1371/journal.pone.0003239 (PMC2528950; doi:10.1371/journal.pone.0003239)
Supplement: Table S2 — Summary of SH and ELW tests for alternative topologies derived from the recombination events detected (0.19 MB PDF) [file pone.0003239.s002.pdf]

One breakpoint events:

NS5A				Trees (nt)		test 1 vs 2				test 2 vs 1			
**Group**	**Patient**	**Event**	**Breakpoint 1**	**1**	**2**	**-lk best**	**%Diff in logLK**	**p-SH**	**p-ELW**	**-lk best**	**%Diff in logLK**	**p-SH**	**p-ELW**
**HCV 0**	**nA28T0**	**1**	**389-416**	1-389	390-743	1049,1100	1,1948	<0,0001	<0,0001	1059,5500	2,8149	<0,0001	<0,0001
	**nC29T0**	**1**	**370-446**	1-370	371-743	726,5000	2,2409	<0,0001	<0,0001	981,5200	3,1991	<0,0001	<0,0001
		**2**	**400-407**	1-400	401-743	786,8100	2,2685	<0,0001	<0,0001	915,3100	2,8254	0,0030	<0,0001
		**3**	**228-237**	1-228	229-743	437,5100	1,7872	<0,0001	<0,0001	1317,0800	4,7344	<0,0001	<0,0001
	**nC35T0**	**1**	**428-437**	1-428	429-743	1600,3600	4,1919	<0,0001	<0,0001	1044,5600	2,9408	<0,0001	<0,0001
		**2**	**369-390**	1-369	370-743	1278,2900	3,9038	<0,0001	<0,0001	1354,1600	4,0380	<0,0001	<0,0001
		**3**	**424-464**	1-424	425-743	1572,5000	4,2385	<0,0001	<0,0001	1065,6300	2,9224	<0,0001	<0,0001
	**nG05T0**	**1**	**576-597**	1-576	597-741	1552,4800	2,5646	<0,0001	<0,0001	390,2300	1,0786	<0,0001	<0,0001
		**2**	**258-276**	1-258	259-741	641,1100	1,0679	<0,0001	<0,0001	1225,6100	3,2048	<0,0001	<0,0001
	**nG07T0**	**1**	**474-475**	1-474	475-741	1292,9800	2,3355	<0,0001	<0,0001	1040,0800	1,7573	<0,0001	<0,0001
	**nG08T0**	**1**	**345-420**	1-345	346-743	772,6800	1,3974	<0,0001	<0,0001	943,8900	2,2543	<0,0001	<0,0001
		**2**	**443-496**	1-443	444-743	1074,8000	1,5792	<0,0001	<0,0001	672,6700	1,5452	<0,0001	<0,0001
**HCV T**	**nC05T1**	**1**	**266-338**	1-266	267-743	695,9200	2,4511	<0,0001	<0,0001	1177,2100	2,6092	<0,0001	<0,0001
		**2**	**341-359**	1-341	342-743	896,8900	2,0425	<0,0001	<0,0001	976,2800	2,1970	<0,0001	<0,0001
	**nG07T2**	**1**	**585-599**	1-585	586-743	2121,7600	3,8772	<0,0001	<0,0001	574,2200	1,8352	<0,0001	<0,0001
		**2**	**390-401**	1-390	391-743	1266,5700	2,7560	<0,0001	<0,0001	1359,7900	3,5283	<0,0001	<0,0001
		**3**	**281-326**	1-281	282-743	916,5600	2,6781	<0,0001	<0,0001	1741,1810	3,8880	<0,0001	<0,0001

E1-E2				Trees (nt)		test 1 vs 2				test 2 vs 1			
**Group**	**Patient**	**Event**	**Breakpoint**	**1**	**2**	**-lk best**	**%Diff in logLK**	**p-SH**	**p-ELW**	**-lk best**	**%Diff in logLK**	**p-SH**	**p-ELW**
**HCV 0-0**	**EC0600**	**2**	**199-200**	1-199	200-534	424,7200	2,1448	<0,0001	<0,0001	1030,8700	1,4607	<0,0001	<0,0001
		**3**	**357-375**	1-357	358-534	961,5000	3,5780	<0,0001	<0,0001	431,5300	0,4253	<0,0001	<0,0001
		**4**	**135-147**	1-135	136-534	257,1200	1,0309	<0,0001	<0,0001	1368,8500	2,7168	<0,0001	<0,0001
	**V035**	**1**	**264-367**	1-265	266-534	791,1000	4,5611	<0,0001	<0,0001	681,8200	3,0000	<0,0001	<0,0001
		**2**	**135-147**	1-135	136-534	258,4600	0,5230	0,0220	0,0180	1358,0700	4,6291	<0,0001	<0,0001
**HCV 0-T**	**C230T**	**1**	**312-351**	1-312	313-534	963,2900	3,7681	<0,0001	<0,0001	555,4800	1,7551	<0,0001	<0,0001
		**2**	**223-231**	1-223	224-534	623,0700	1,9067	<0,0001	<0,0001	911,8400	2,1119	0,0010	<0,0001
	**C300T**	**2**	**242-320**	1-242	243-531	654,0300	1,6379	<0,0001	<0,0001	866,6900	1,8718	<0,0001	<0,0001
	**C5703**	**1**	**264-285**	1-264	265-531	794,7300	1,9083	<0,0001	<0,0001	631,8200	0,7926	<0,0001	<0,0001
**HCV 0**	**A16T0**	**2**	**264-295**	1-264	265-471	1860,5400	12,8531	<0,0001	<0,0001	1098,6000	4,4094	<0,0001	<0,0001
	**A21T0**	**2**	**211-214**	1-211	212-471	836,3600	2,4001	<0,0001	<0,0001	1245,1100	3,2024	<0,0001	<0,0001
	**A28T0**	**1**	**258-291**	1-258	259-471	1267,6400	12,1686	<0,0001	<0,0001	1534,0900	4,5292	<0,0001	<0,0001
		**2**	**258-270**	1-258	259-471	1267,6400	12,1686	<0,0001	<0,0001	1534,0900	4,5292	<0,0001	<0,0001
	**C28T0**	**1**	**233-242**	1-233	234-471	1013,8400	3,8935	<0,0001	<0,0001	1084,9600	4,7759	<0,0001	<0,0001
		**2**	**250-276**	1-250	251-471	119,7400	5,9621	<0,0001	<0,0001	933,6700	4,3859	<0,0001	<0,0001
	**C29T0**	**1**	**223-229**	1-223	224-477	598,8500	2,2007	<0,0001	<0,0001	616,1100	3,2204	<0,0001	<0,0001
		**2**	**232-258**	1-232	233-477	611,9800	2,1235	<0,0001	<0,0001	944,7700	3,4150	<0,0001	<0,0001
	**C32T0**	**2**	**232-242**	1-232	233-471	992,2300	5,1973	<0,0001	<0,0001	1397,9600	4,0448	<0,0001	<0,0001
		**3**	**249-282**	1-249	250-471	1126,3400	5,2045	<0,0001	<0,0001	876,0300	3,8205	<0,0001	<0,0001
		**4**	**230-249**	1-230	231-471	970,4000	4,3072	<0,0001	<0,0001	1034,5100	4,2798	<0,0001	<0,0001
	**G08T0**	**1**	**249-285**	1-249	250-471	1331,2000	12,2327	<0,0001	<0,0001	1255,0800	8,0499	<0,0001	<0,0001
		**2**	**285-294**	1-285	286-471	1518,1700	12,6229	<0,0001	<0,0001	1096,3800	8,0720	<0,0001	<0,0001
		**3**	**289-294**	1-289	290-471	1556,8800	12,7090	<0,0001	<0,0001	1060,8700	7,7520	<0,0001	<0,0001
**HCV T**	**A21T1**	**2**	**211-215**	1-211	212-472	1277,4100	4,3944	<0,0001	<0,0001	2222,0800	5,6507	<0,0001	<0,0001
	**G17T1**	**2**	**313-322**	1-313	314-472	1289,2500	10,1701	<0,0001	<0,0001	635,3900	4,0986	<0,0001	<0,0001
	**G16T1**	**1**	**325-354**	1-325	326-480	1459,2200	6,0599	<0,0001	<0,0001	718,3500	3,4711	<0,0001	<0,0001
		**2**	**234-247**	1-234	235-480	972,4400	4,3007	<0,0001	<0,0001	1175,0800	4,6153	<0,0001	<0,0001
		**3**	**285-319**	1-285	286-480	1230,0100	1,0780	<0,0001	<0,0001	895,1800	4,1196	0,0010	<0,0001
	**eG26T2**	**1**	**301-333**	1-301	302-472	989,5500	4,0999	<0,0001	<0,0001	491,1000	1,4022	0,0030	<0,0001

Two samples				Trees (nt)		test 1 vs 2				test 2 vs 1			
**Group**	**Patient**	**Event**	**Breakpoint**	**1**	**2**	**-lk best**	**%Diff in logLK**	**p-SH**	**p-ELW**	**-lk best**	**%Diff in logLK**	**p-SH**	**p-ELW**
**NS5a(T0-T1)**	**nC29T0-T1**	**1**	**370-407**	1-370	371-743	830,0300	3,1687	<0,0001	<0,0001	1116,3300	5,3226	<0,0001	<0,0001
		**2**	**228-237**	1-228	229-743	521,7100	3,3323	<0,0001	<0,0001	1469,9800	7,0503	<0,0001	<0,0001
		**3**	**297-329**	1-297	298-743	655,8600	3,6121	<0,0001	<0,0001	1312,6100	5,6075	<0,0001	<0,0001
		**4**	**370-446**	1-370	371-743	829,6900	3,5654	<0,0001	<0,0001	1115,4600	5,3266	<0,0001	<0,0001
	**nG07T0-T2**	**1**	**258-327**	1-258	259-741	1592,9500	76,3234	<0,0001	<0,0001	2358,2500	8,8285	<0,0001	<0,0001
		**5**	**421-447**	1-421	422-741	1767,3300	6,1794	<0,0001	<0,0001	1596,6600	6,8207	<0,0001	<0,0001
		**6**	**105-114**	1-105	160-741	435,9500	2,6969	<0,0001	<0,0001	3138,0400	8,9886	<0,0001	<0,0001
		**8**	**225-327**	1-225	226-741	940,6900	5,3209	<0,0001	<0,0001	2464,7700	8,6814	<0,0001	<0,0001
		**9**	**421-439**	1-421	422-741	1592,9500	5,3617	<0,0001	<0,0001	1998,3300	6,5172	<0,0001	<0,0001
**E1-E2(T0-T1)**	**eA21T0-T1**	**1**	**210-214**	1-210	211-471	2242,5100	9,4682	<0,0001	<0,0001	2242,5100	9,4682	<0,0001	<0,0001
	**eC29T0-T1**	**2**	**318-321**	1-318	319-477	1027,6400	9,8595	<0,0001	<0,0001	482,3400	2,9946	<0,0001	<0,0001
	**eG16T0-T1**	**1**	**234-247**	1-234	235-480	1016,4000	4,3796	<0,0001	<0,0001	1312,5600	4,3921	<0,0001	<0,0001
		**2**	**259-265**	1-259	260-480	1128,4400	4,8346	<0,0001	<0,0001	1206,9600	4,5046	<0,0001	<0,0001
	**eG26T0-T1-T2**	**1**	**301-333**	1-301	302-472	1023,7300	3,5151	0,0010	0,0010	555,9400	2,0557	0,0010	<0,0001

Two breakpoints events:

NS5A					Trees (nt)			test 2 vs 1				test 2 vs 3		
**Group**	**Patient**	**Event**	**Breakpoint 1**	**Breakpoint 2**	**1**	**2**	**3**	**-lk best**	**%Diff in logLK**	**p-SH**	**p-ELW**	**%Diff in logLK**	**p-SH**	**p-ELW**
**HCV 0**	**nC36T0**	**1**	**74-89**	**356-418**	1-88	89-356	357-743	842,3100	3,8954	<0,0001	<0,0001	2,7645	<0,0001	<0,0001
	**nG07T0**	**2**	**258-327**	**600-616**	1-326	327-600	601-741	855,7500	1,7840	<0,0001	<0,0001	2,3018	<0,0001	<0,0001
**HCV T**	**nG07T2tfa**	**4**	**59-80**	**390-423**	1-79	80-390	391-743	945,7900	2,0402	0,0010	<0,0001	1,2180	<0,0001	<0,0001

E1E2					Trees (nt)			test 2 vs 1				test 2 vs 3		
**Group**	**Patient**	**Event**	**Breakpoint 1**	**Breakpoint 2**	**1**	**2**	**3**	**-lk best**	**%Diff in logLK**	**p-SH**	**p-ELW**	**%Diff in logLK**	**p-SH**	**p-ELW**
**HCV 0-0**	**EC0600**	**1**	**165-177**	**306-331**	1-176	177-306	307-534	346,8700	0,3023	<0,0001	<0,0001	0,3243	<0,0001	<0,0001
**HCV 0-T**	**C300T**	**1**	**242-261**	**450-477**	1-260	261-450	451-531	490,3900	1,8263	<0,0001	<0,0001	1,6614	<0,0001	<0,0001
**HCV 0**	**A16T0**	**1**	**176-190**	**348-351**	1-189	190-348	349-471	1050,2700	12,1583	<0,0001	<0,0001	6,0974	<0,0001	<0,0001
	**A21T0**	**1**	**191-196**	**294-343**	1-195	196-294	295-471	477,6700	4,4200	<0,0001	<0,0001	2,9560	<0,0001	<0,0001
	**C32T0**	**1**	**42-162**	**264-282**	1-161	162-264	265-471	577,4900	4,1762	<0,0001	<0,0001	8,3820	<0,0001	<0,0001
	**G08T0**	**4**	**81-112**	**249-285**	1-111	112-249	250-471	859,6600	10,2248	<0,0001	<0,0001	9,7831	<0,0001	<0,0001
**HCV T**	**A21T1**	**1**	**157-170**	**250-277**	1-169	170-250	251-472	631,2500	7,2510	<0,0001	<0,0001	7,8971	<0,0001	<0,0001
	**G17T1**	**1**	**177-191**	**313-322**	1-191	191-313	314-472	469,3000	6,8658	0,0010	<0,0001	2,4021	<0,0001	<0,0001
		**3**	**178-198**	**250-259**	1-197	198-250	251-472	223,4500	4,4790	<0,0001	<0,0001	1,9923	0,0010	<0,0001

Two Samples			Trees (nt)		Trees (nt)			test 2 vs 1				test 2 vs 3		
**Group**	**Patient**	**Event**	**Breakpoint 1**	**Breakpoint 2**	**1**	**2**	**3**	**-lk best**	**%Diff in logLK**	**p-SH**	**p-ELW**	**%Diff in logLK**	**p-SH**	**p-ELW**
**NS5a(T0-T1)**	**nG07T0-T2**	**2**	**258-327**	**600-616**	1-258	259-615	616-741	1558,2700	6,1082	<0,0001	<0,0001	4,6382	<0,0001	<0,0001
		**3**	**327-336**	**547-573**	1-327	328-572	573-741	1414,8800	4,5397	<0,0001	<0,0001	3,6425	<0,0001	<0,0001
		**4**	**258-327**	**480-491**	1-258	259-490	491-741	873,6700	3,3578	<0,0001	<0,0001	2,4776	<0,0001	<0,0001
		**7**	**105-114**	**547-573**	1-105	106-572	573-741	1998,3300	6,5172	<0,0001	<0,0001	4,9738	<0,0001	<0,0001
