# Supplementary material for: Evidence of Recombination in Intrapatient Populations of Hepatitis C Virus
Source: PLoS One. 2008 Sep 18;3(9):e3239. doi: 10.1371/journal.pone.0003239 (PMC2528950; doi:10.1371/journal.pone.0003239)
Supplement: Table S3 — Summary of Mann-Whitney tests for differences in recombination frequency between clinical groups considered in this study (0.09 MB DOC) [file pone.0003239.s003.doc]

Supporting Information S3

Summary of Mann-Whitney tests for the comparisons between different groups with respect to frequency of detected recombination events in HCV regions E1-E2 and NS5A.

HIV treatment effect

HCV Group	N	Average Range	Sum of ranges	Z	Asymptotic sig. (bilateral)
E1E2 HCV 0-0	16	16,5625	265,00	-0,4058	0,6848 **ns**
E1E2 HCV 0-T	17	17,4118	296,00		
Total	33				

HIV co-infection effect

HCV Group	N	Average Range	Sum of ranges	Z	Asymptotic sig. (bilateral)
E1E2 HCV 0-0	16	48,3125	773,00	-0,4174	0,6763 **ns**
E1E2 HCV 0	77	46,7273	3598,00		
Total	93				

E1-E2 HCV treatment effect

HCV Group	N	Average Range	Sum of ranges	Z	Asymptotic sig. (bilateral)
E1E2 HCV 0	77	51,1818	3941,00	-0,8940	0,3713 **ns**
E1E2 HCV T	26	54,4231	1415,00		
Total	103				

NS5A HCV treatment effect

HCV Group	N	Average Range	Sum of ranges	Z	Asymptotic sig. (bilateral)
NS5a HCV 0	73	49,6986	3628,00	-0,2362	0,8132 **ns**
NS5a HCV T	25	48,9200	1223,00		
Total	98				

HCV region effect

HCV Group	N	Average Range	Sum of ranges	Z	Asymptotic sig. (bilateral)
E1E2 HCV 0	77	75,3200	5799,50	-0,1040	0,917 **ns**
NS5A HCV 0	73	75,6900	5525,50		
Total	150				

HCV region treatment effect

HCV Group	N	Average Range	Sum of ranges	Z	Asymptotic sig. (bilateral)
E1E2 HCV T	26	26,9200	700,00	-0,8100	0,418 **ns**
NS5A HCV T	25	25,0400	626,00		
Total	51				

Mann-Whitney test p-value =0.05
